# Interplay of bimolecular and Auger recombination in photoexcited carrier dynamics in silicon nanocrystal/silicon dioxide superlattices

**DOI:** 10.1038/s41598-018-19967-x

**Published:** 2018-01-26

**Authors:** T. Chlouba, F. Trojánek, J. Laube, D. Hiller, S. Gutsch, M. Zacharias, P. Malý

**Affiliations:** 1 Department of chemical physics and optics, Faculty of Mathematics and Physics, Charles University, Prague, 12116 Czech Republic; 2 IMTEK, Faculty of Engineering, Albert-Ludwigs-University Freiburg, Georges-Köhler-Alle 103, 79110 Freiburg, Germany

## Abstract

We report results of investigating carrier recombination in silicon nanocrystal/silicon dioxide superlattices. The superlattices prepared by nitrogen-free plasma enhanced chemical vapour deposition contained layers of silicon nanocrystals. Femtosecond transient transmission optical spectroscopy was used to monitor carrier mechanisms in the samples. The three-particle Auger recombination was observed in accord with previous reports. However, under high pump intensities (high photoexcited carrier densities) the bimolecular process dominated the recombination. Detailed analysis of measured data and fitting procedure made it possible to follow and quantify the interplay between the two recombination processes. The bimolecular recombination was interpreted in terms of the trap-assisted Auger recombination.

## Introduction

Understanding carrier recombination processes in semiconductor materials and nanostructures is of fundamental importance because they determine the electron dynamics and quantum efficiency in a number of devices. The distinct recombination processes are known and described well on a microscopic level however the recombination mechanisms limiting the carrier lifetime in specific structures or devices are in general ambiguous. This is also the case of silicon nanocrystals investigated intensely over the last two decades. An important nonradiative recombination mechanism is the Auger process in which two charge carriers recombine giving the released energy to the third carrier which is excited into a higher energy state fulfilling the momentum conservation law. This Coulomb interaction-mediated process reported in bulk semiconductors as well as in semiconductor quantum structures can be intrinsic or extrinsic, i.e. defect-related. It dominates under high carrier densities in bulk materials but it is also very important in semiconductor nanocrystals where a small number of photoexcited carriers within a nanometer-sized volume correspond to bulk high densities. The Auger recombination in nanocrystals is affected by the presence of a real crystal surface and surrounding medium, and first of all by quantum confinement (an uncertainty in quasimomentum). If the confinement energy is larger than the exciton binding energy the charge carriers are uncorrelated^1^. Auger recombination follows similar dependence as in the bulk material with a modified coefficient, though^2–5^. In case of small confinement energy at least in one direction like in quantum wires, the excitons take part in the Auger recombination. The Auger recombination of excitons is also typical of molecular materials^6^ and of carbon nanotubes^7^. Defect-related Auger recombination where one of the three interacting carriers is trapped has been observed in heavily doped semiconductors^8^ and also in semiconductor nanocrystals^9^.

The recombination processes are measured usually by monitoring the dynamics of population of charge carriers after photoexcitation by short light pulses using time-resolved transient absorption (pump and probe) or luminescence techniques. Most of the research has been done on CdSe and silicon nanocrystals^2,3,5,9–14^. The experimental data have been analysed by models based on rate equations describing the whole carrier population or on rate equations describing the recombination of carriers in sets of nanocrystals containing initially different, statistically distributed, carrier numbers^3^.

The dynamics of photoexcited nonequilibrium carriers due to Auger recombination can be described well by the rate equation

1 $$\frac{dN(t)}{dt}=-C{N}^{3}$$

where *N* is the carrier density and C is the Auger constant. This equation applies for bulk semiconductors and nanocrystals in case of uncorrelated carriers (this is often supposed to be the “proper” Auger recombination). On the other hand, the equation

2 $$\frac{dN(t)}{dt}=-B{N}^{2}$$

applies for the excitonic Auger recombination with B being the bimolecular recombination constant. The latter type of the decay is often called bimolecular decay. The bimolecular decay is typical also of the extrinsic Auger process

3 $$\frac{dN(t)}{dt}=-C{N}_{t}{N}^{2},$$

where N_t_ is density of defects taking part in the process. Even though the Auger process is a many-body effect usually the coefficients are assumed to be independent of carrier populations. Fitting e.g. the photoluminescence or transient transmission decay curves by eqs (1–3) can provide the coefficient value.

In this paper we report on results of an experimental study of carrier dynamics in arrays of silicon nanocrystals in silicon dioxide matrix which indicate that the photoexcited carrier recombination has features of the Auger process (three particle) which is dominated by a bimolecular process at high pump intensities, i.e. a large number of photoexcited carriers per nanocrystal.

## Experimental Details

**Figure 1:**
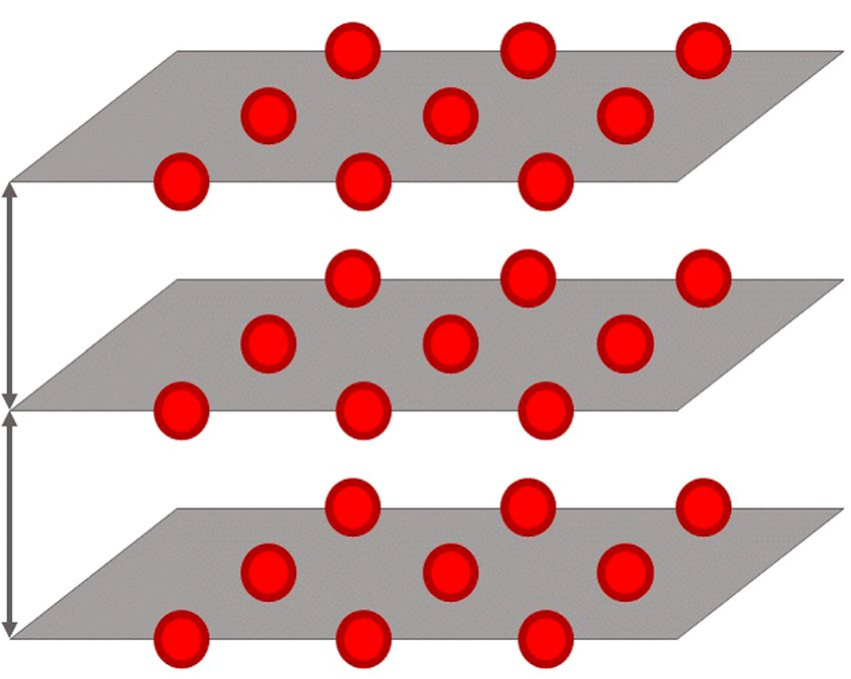
Ilustration of the structure of samples. Positions of nanocrystals are randomized within the 2D structures of SiO_x_ layers yet they do not intrude into SiO_2_ surrounding layers.

The samples were prepared by deposition of 100 SiO_x_/SiO_2_ bilayers onto a quartz glass substrate by nitrogen free SiH_4_ + O_2_ PECVD process^15^. The thickness of SiO_2_ barrier is 4 nm and SiO_x_ layer thicknesses are 4.5 nm for all samples. There are five samples with different stochiometric parameter x with x = {0.3, 0.5, 0.7, 0.9, 1.3}. These samples were subsequently annealed at 1100 °C for 1 h in high-purity N_2_ which produces Si nanocrystals within SiO_2_ matrix^16^. The position of nanocrystals is randomized within the SiO_x_ layers but they do not intrude into SiO_2_ layers. The mean diameter of nanocrystals should be then mostly determined by SiO_x_ layer width. The structure is illustrated in Fig. 1. One batch of these samples was additionaly post-annealed at 500 °C for 1 h in high-purity H_2_ to passivate Si dangling bond defects^17^.

Since growth parameters are well known, we used similar approach to^17^ and calculated excess silicon in samples. We estimated nanocrystal size and densities mostly from TEM measurements^18^ of similar samples (prepared by similar procedure) albeit the distribution of sizes is relatively large. Calculated and estimated quantities for our samples are in Table 1.

**Table 1:** Properties of measured samples.

Label	x = 0.3	x = 0.5	x = 0.7	x = 0.9	x = 1.3
x	0.3	0.5	0.7	0.9	1.3
Nanocrystal areal density/cm^−2^	10^13^*	1.2 · 10^14^*	1.5 · 10^14^	2.1 · 10^14^	(1.5 · 10^14^)
Average NC diameter/nm	17^*^	7^*^	5	3.3	(3)
Distance to nearest neighbor (in layers)/nm	*	*	1.3	2	(2.4+)
Transmission of sample (at 400 nm)/%	5	42	79	85	87
Excess silicon/%	27	21	16	13	7

Each sample consists of 100 bilayers of SiO_x_/SiO_2_. Average nanocrystal diameter for samples x = 0.7, x = 0.9 is estimated from TEM measurements of similar samples and is burdened by relatively large dispersion^18^. The nanocrystal areal density and nearest neighbor distance are also estimated from TEM measurements. Samples x = 0.3 and x = 0.5 (*) are strongly percolated therefore it is meaningless to talk about distance to nearest neighbor. Also the average NC diameter (*) for these two samples was estimated from terahertz spectroscopy and relate to silicon objects (clusters, nanocrystals) which participate in conductivity (absorption)^28^. Nanocrystal areal density, diameter and distance to nearest neighbor for sample x = 1.3 (in parentheses) are estimated based on excess silicon calculations and other samples TEM measurements.

We employed a standard pump and probe (transient transmission) setup with 1 kHz repetition rate laser system (Tsunami, Spitfire, Newport) with 100 fs pulse length. The fundamental 800 nm laser output was frequency doubled to 400 nm to achieve excitation above the absorption edge of silicon nanocrystals. Probe pulse (800 nm) energy was detected by a silicon detector behind the sample. Part of the probe beam was split of before the sample and focused into a reference detector. Electrical signal from the reference detector was subtracted from the main detector signal and difference signal was then pre-amplified before being fed into a lock-in amplifier. This setup allows us to increase the sensitivity of detection, decrease electronical noise and also suppresses laser fluctuations. The probe beam fluence was kept constant at about 15 *μ*J/cm^2^. Excitation (pump) beam fluence was varied by neutral filters from 2.1 to 16.5 mJ/cm^2^. All measurements were done at room temperature.

By measuring the pump intensity before the sample and reflected and transmitted pump intensity we estimated the volume electron-hole pair density in silicon and average number of excited electron-hole pairs per nanocrystal. While the number of electron-hole pairs per nanocrystals may be burdened by large dispersion of nanocrystal sizes/density, the volume density is not dependent on nanocrystal size and therefore is much more reliable quantity.

## Results and Discussion

**Figure 2:**
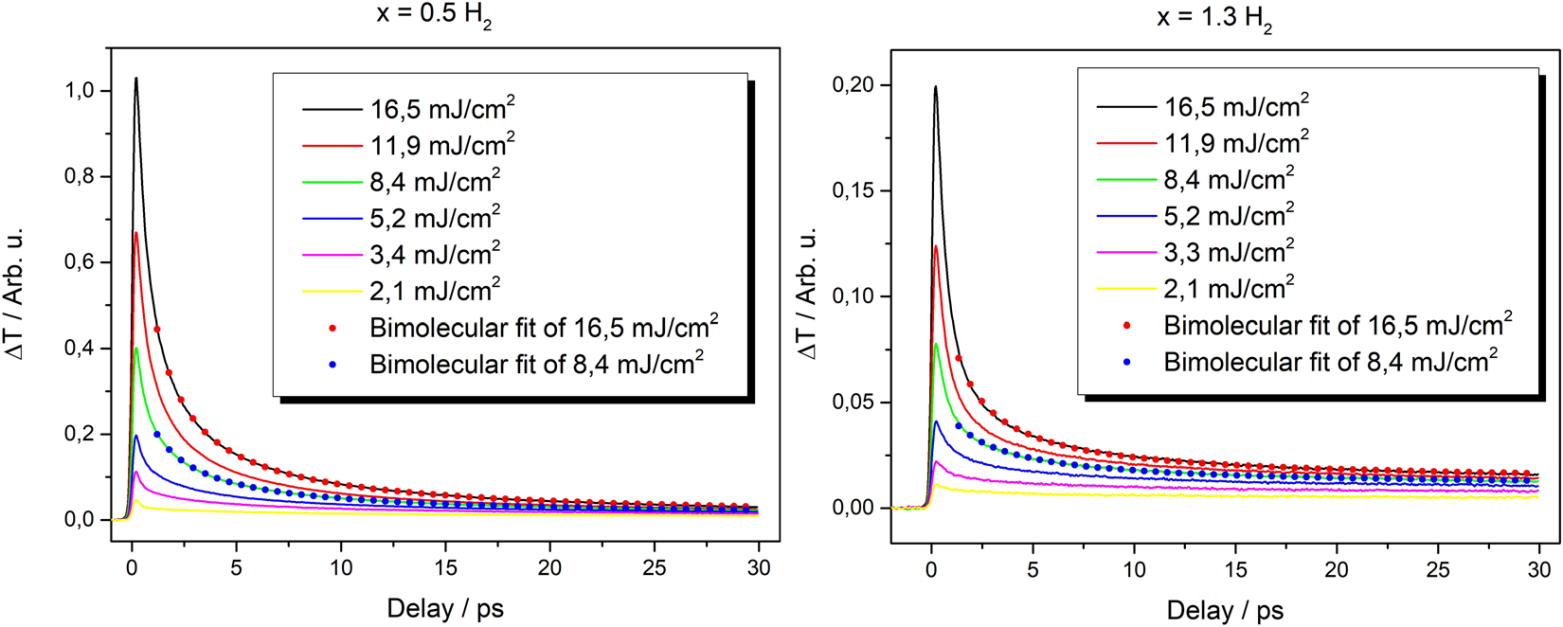
Intensity dependence of transient transmission dynamics for sample x = 1.3 and x = 0.5. Both samples were additionally annealed in hydrogen. Red and blue dots are bimolecular fits of associated dynamics.

Results of transient transmission measurements for two selected samples (x = 1.3 and x = 0.5) are shown in Fig. 2. The transient transmission signals are positive indicating an induced absorption as is expected^19^. There is a very small probability of indirect absorption of 800 nm (1.55 eV) photons by valence band-conduction band transition from *Γ* point to *X* valleys^20^ and intra-band excited carrier absorption (by previously excited electrons in *X* valleys and holes) dominates. The absorption at 800 nm is thus supposed to be proportional to the density of photoexcited carriers^21^. Dynamics of transient transmission are shown for selected samples for different excitation levels in Fig. 2. The peak value of the signal increases and the decay gets faster with increasing pump fluence.

**Figure 3:**
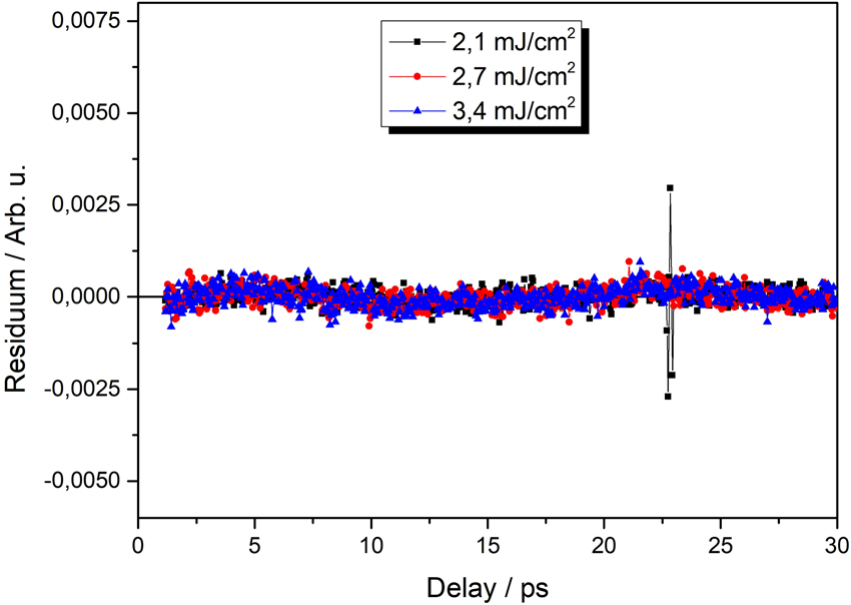
Residua of Auger recombination fits for sample x = 0.5 (H-passivated) for low fluences. Arbitrary units are of the same scale as in Fig. 2.

**Figure 4:**
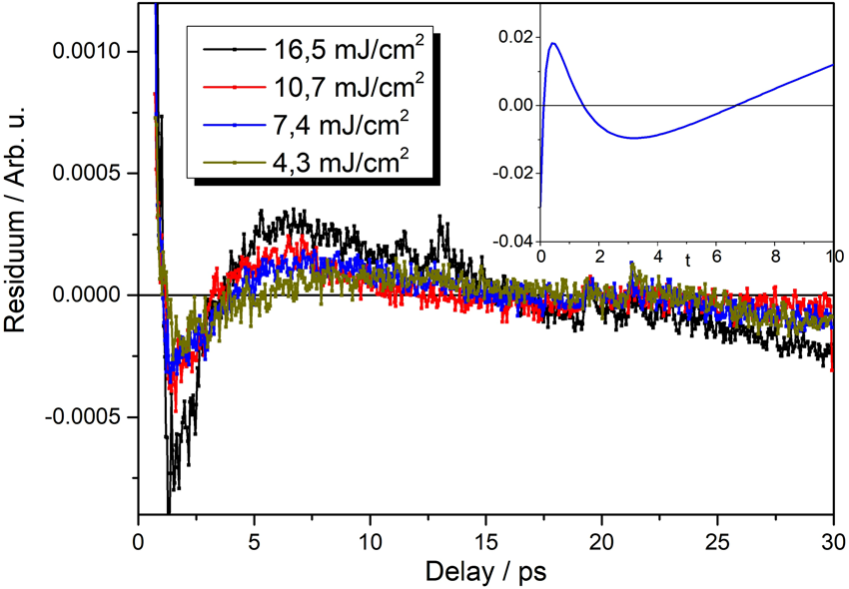
Residua of bimolecular fits for sample x = 0.5 (H-passivated) for high fluences. Arbitrary units are of the same scale as in Fig. 2. Inset depicts the difference Auger – bimolecular function with parameters: B = N_0_ = k = 1, y_0_ = 0 for bimolecular decay, C = 1, N_0_ = 1.08, k = 1.1, y_0_ = −0.16 for Auger decay resulting in function $(\frac{1,1}{\sqrt{2.33\,\,{t}+1}}-\frac{1}{{t}+1}-0.16)$. This function has the same shape (up to a sign) as bimolecular fit residuals which implies that there is Auger recombination component present in high fluence decay curves.

Different pump fluences lead to different initial photoexcited carrier densities. According Eqs 1–3 this should cause changes in both the amplitude and shape of the decay. The analysis of the experimental data using these equations can thus reveal the type of dominating recombination process and also the values of the relevant (pump intensity independent) coefficients. Because of influence of finite laser pulse width and fast initial thermalization processes, we focus on data from about 1 ps after the maximum signal^14^. Equation (1) corresponds to a three-particle Auger process as described in the introduction. In this case the measured signal *S* can be fitted by the following function

4 $$S(t)=kN(t)=\frac{k{N}_{0}}{\sqrt{2{N}_{0}^{2}Ct+1}}+{y}_{0},$$

which is solution of Eq. (1) with addition of a constant backround which represents the decay rates slower than hundreds picoseconds (carriers in nanocrystals with a single electron-hole pair, carriers in long-lived traps). Here *N*_0_ is initial carrier density at time t = 0, *k* is an experimental constant. We have found that this function can fit the data very well in case of lower excitation fluences. For higher pump fluences, however, the fitting leads to unrealistic values of fitting parameters or fails completely. On the contrary, in this high fluence regime, the signals can be described well by the solution of Eq. (2)

5 $$S(t)=kN(t)=\frac{k{N}_{0}}{{N}_{0}Bt+1}+{y}_{0},$$

to which the constant y_0_ was added again. A bimolecular recombination process thus dominates the decay for high pump fluences. This implies that both types – proper Auger and bimolecular recombination can take place in our samples. A detailed investigation of the fitting procedure provides more details. The residua of the fits of low-pump-power data by Eq. (4) are symmetric around zero within experimental error (which is about 1% of the signal) indicating that three-particle Auger process with addition of a long living component describe very well the measured dynamics (see Fig. 3). On the other hand, fiting of the high-power-data with Eq. (5) results in strongly asymmetric residua with the characteristic shape (see Fig. 4). It can be shown that this shape results from attempting to fit a decay function of Eq. (4) by Eq. (5) (see the inset of Fig. 4). At the same time, the data cannot be fitted well or, if at all, only by Eq. (4). This means that while the dominant process governing the transient transmission dynamics at high pump powers is certainly bimolecular there is also an underlying presence of Auger recombination at all fluences. The analysis of measured data by fitting can thus provide information on the “transition” pump fluence when the bimolecular process starts to dominate the carrier recombination. The fitting also provides the constants B and C of the Auger and bimolecular recombination respectively.

We can try to fit the bimolecular recombination curve (Eq. (5)) by the Auger recombination (Eq. (4)). This can be done in principle but it will unsurprisingly result in different values for fitting parameters. We assume a bimolecular function with simple parameters B = N_0_ = k = 1, y_0_ = 0, e.g. $\frac{1}{1+t}$. If we try to fit this function with the Auger function over the range $t\in (0,10)$ we will end up with parameters for Auger decay (assuming C = 1) N_0_ = 1.08, k = 1.1, y_0_ = −0.16. This is just a toy model, however some general observations can be made. First, the residuum from such fitting will have characteristic shape as depicted in the inset of Fig. 4. The opposite process, fitting of Auger decay function by bimolecular decay function will result in the same residuum shape up to a sign. Second, fitting bimolecular decay with Auger decay function will always result in either smaller fitted y_0_ or even negative y_0_. Fitted (Auger) N_0_ will always be higher than bimolecular N_0_ even more profoundly than here because C is much smaller than B in real samples. We can use these mathematical properties to determine the dominant recombination process and transition fluence.

Parameters B and C for each sample can be determined from fitting the data measured under the highest and lowest pump fluence. The initial carrier density N_0_ was estimated using the procedure described in^13^. For intermediate pump powers, we fix the B and C and our fitting parameters are N_0_ and y_0_. The reason why we let N_0_ vary is that its value is one of the criteria used for determining the transition fluence (e.g. where Auger function fails).

In a search for the transition fluence we have three criteria for determining when the dominating process is bimolecular instead of Auger (while fitting with Auger function).

1. Disctinctively larger fitted N_0_ than theoreticaly predicted
2. Negative fitted y_0_
3. Pronounced nonsymmetric residuum with shape similar to Fig. 4.

When at least two of these three conditions are satisfied we conclude that the decay curve is mainly governed by bimolecular recombination.

**Figure 5:**
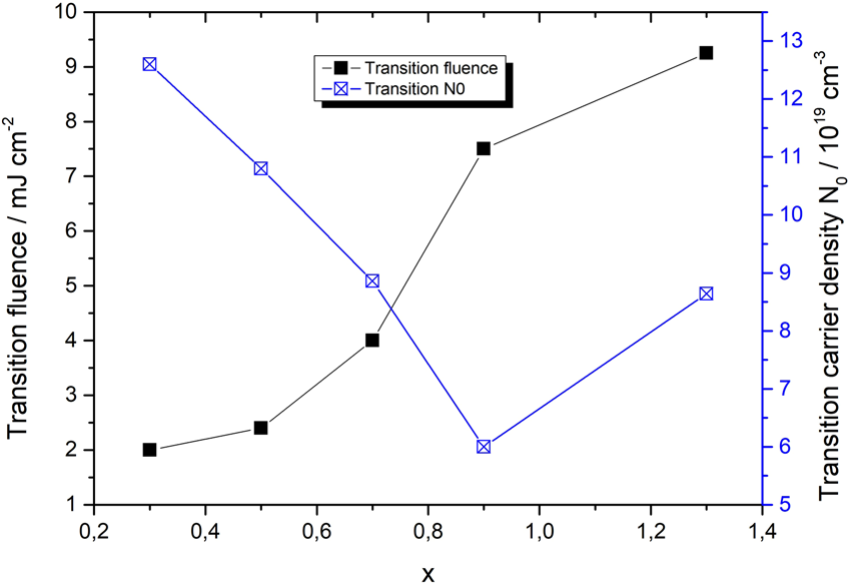
Transition fluence and transition initial carrier density in dependence on stoichiometric parameter x. All samples were annealed in hydrogen.

The values of the transition fluence, i.e. fluence when the carrier dynamics start to be dominated by the bimolecular type of the decay found by the analysis described above, are plotted in Fig. 5 for different values of stoichiometric parameter *x*. The corresponding estimated volume carrier densities in dependence on sample stochiometric parameter x. While the transition fluence increases with x the (more fundamental) transition carrier density decreases (due to transmittance and excess silicon). Perhaps, the even more important parameter is the number of electron-hole pairs per nanocrystal but this is influenced by large errors of determining nanocrystal sizes. Neverthless the transition number of carriers per nanocrystal is roughly constant for nonpercolated nanocrystals, cf. Fig. 5.

**Figure 6:**
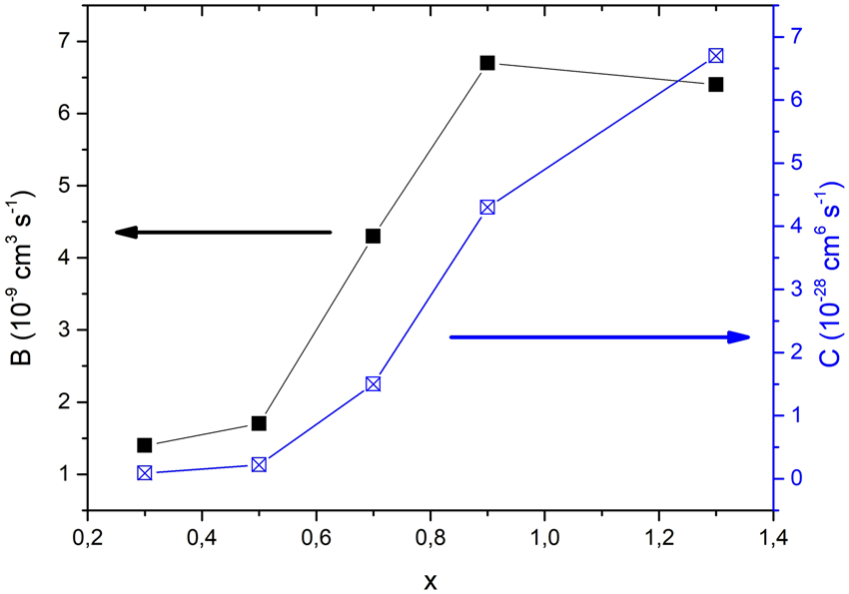
Coefficients B and C for bimolecular and Auger recombination in dependence on stoichiometric parameter x. The coefficient B is varying within one order of magnitude while parameter C is encompassing over two orders of magnitude. All samples were post-annealed in hydrogen for defect passivation.

In Fig. 6 we plot recombination coefficients *C* and *B* for every sample. These constants are of the same order as those in similar semiconductor systems^22–24^ and are both increasing with increasing x. The *C* coefficient grows much faster over two orders of magnitude. The *C* and *B* growth can be interpreted in terms of shrinking of nanocrystal sizes with increasing parameter *x* as observed in TEM measurements in^18^. Both bimolecular and Auger recombination rates depend on nanocrystal sizes (diameters D) albeit differently^9,25^.

In the past bimolecular recombination was observed in CdSe quantum wires^1,26,27^. The reason for this was that the carriers in a wire can form 1D excitons which then interact mainly by exciton-exciton interaction while in 0D structures the carriers are uncorrelated when there are more than one electron-hole pairs. Our samples with higher silicon volume (x = 0.3, x = 0.5) are indeed somewhat percolated as shown in terahertz conductivity measurements^28^ however samples with lower silicon volume (x = 1.3) with well separated nanocrystals should not show a bimolecular recombination at all. Yet not only bimolecular recombination is observed in these samples, but the relevant recombination coefficient *B* is three to four times higher than in percolated samples. This implies that percolation of nanocrystals cannot explain occurence of bimolecular recombination.

The most feasible possibility is three-particle Auger recombination when one carrier is trapped^9,12,29^. This would require a presence of traps within a silicon band gap. For one particular value of x = 0.7 we measured two samples. One was annealed in H_2_ as any other while one was not annealed. The H_2_ should passivate some of these traps. There is an interesting difference between these two samples. The non-passivated sample has a slightly (+/− 0.5 mJ/cm^2^) lower transition fluency. Also the B parameter is about 40% higher and C is 20% smaller for the non-passivated sample relative to the passivated one. We can estimate the density of these traps using B and C parameters we obtained from fitting (assuming C is almost the same for both Auger processes).

6 $$C{N}^{3}=C{N}^{2}{N}_{trap}=B{N}^{2}$$

This implies the density of traps (for silicon volume) to be of the order of 10^19^ cm^−3^. The density of nanocrystals is V^−1^ ~ 10^19^ cm^−3^ (V being nanocrystal volume) which roughly corresponds to about 1 trap per nanocrystal which is realistic.

This however does not explain the fact that the bimolecular process appears only at high pump levels. Our tentative explanation of that phenomenon is that the traps taking part in trapped carrier Auger recombination are either high in SiO_2_ band gap and therefore have to be filled by preceding free carrier Auger process or their ground state in Si band gap cannot be filled directly (due to selection rules) and has to be filled from its excited state (high above Si conduction band minima) again through Auger process. This can explain both the power dependence and switching of bimolecular recombination and the influence of hydrogen annealing. The exact nature of these traps is not clear but there is evidence of presence of E’ class of defects (which in some cases serve as electron traps) in these samples that are passivated by hydrogen^30^ as well as other types of defects.

## Conclusions

We studied silicon nanocrystals embedded in SiO_2_ dielectric matrix prepared by superlattice technique using ultrafast transient transmission method. While for low fluences the decay of induced absorption clearly reflects the Auger recombination at higher fluences the dominant recombination mechanism is bimolecular. However the three-particle Auger recombination is still present and is clearly visible from fit residuals. There is a fluence transition threshold where bimolecular process becomes dominant. The position of this treshold most likely depends mainly on nanocrystal size and in terms of number of carriers per nanocrystals is roughly constant in our nonpercolated samples. Also this treshold can be slightly elevated by additional hydrogen annealing of the sample. Our explanation of bimolecular recombination is an activation of trapped carrier Auger recombination. Corresponding traps are activated by preceeding Auger effect or free carrier absorption of 400 nm pulse which explains the power dependence. The dependence of recombination properties on stochiometric parameters and nanocrystal diameters should be taken into account while designing devices based on this technology.

### Data avaiability statement

The datasets generated and analysed during the current study are available from the corresponding author on reasonable request.
